# Post-cardiac arrest evaluation: understanding non-shockable rhythms

**DOI:** 10.1093/eurheartj/ehz504

**Published:** 2019-08-13

**Authors:** Matthew C Hyman, Rajat Deo

**Affiliations:** Electrophysiology Section, Division of Cardiovascular Medicine, Perelman School of Medicine at the University of Pennsylvania, Philadelphia, PA, USA

## Abstract

**Figure:**
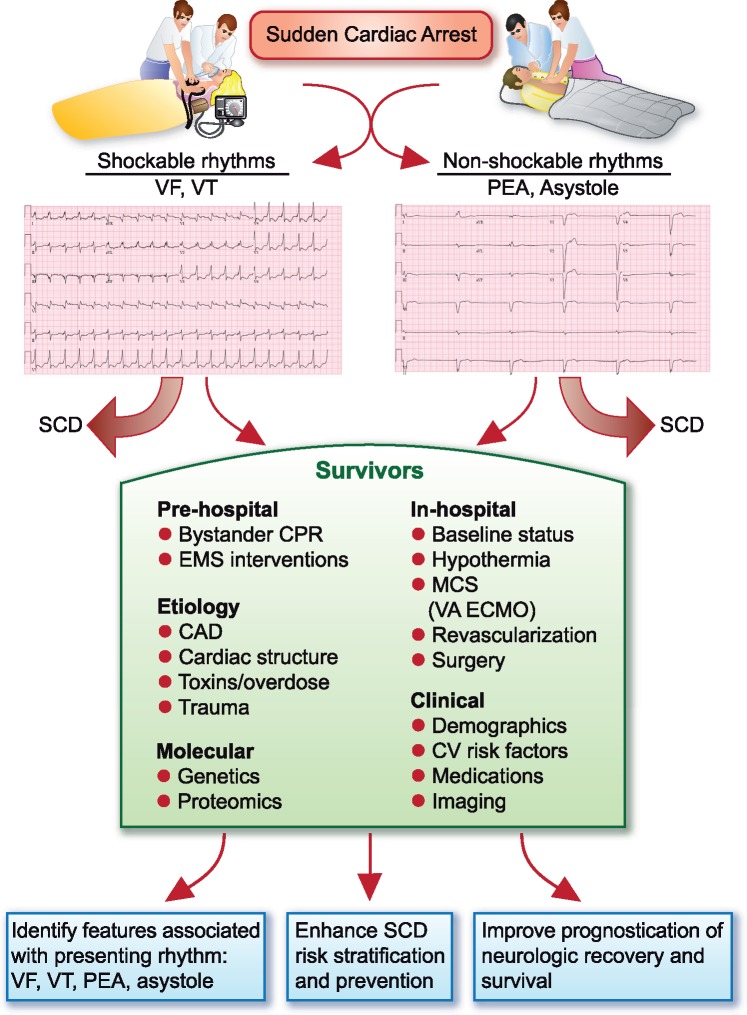

**This editorial refers to ‘Women have lower chances than men to be resuscitated and survive out-of-hospital cardiac arrest’^†^, by M. T. Blom *et al*., on page 3824.**

Sudden cardiac death (SCD) remains a major public heath epidemic worldwide. The recognition of ventricular tachycardia (VT) and ventricular fibrillation (VF) as arrhythmic complications of ischaemia and infarction has resulted in early coronary revascularization and widespread use of beta-blockers, statins, and renin–angiotensin–aldosterone inhibitors. These therapies not only prevent adverse cardiac remodelling, but they also reduce arrhythmic events. Other clinical trials have further evaluated high-risk populations such as those with heart failure and systolic dysfunction for the prevention of SCD. These studies have led to widespread use of the implantable cardioverter defibrillator (ICD) for the rapid termination of VT and VF in high-risk populations. As a result of this progress and ensuing public health campaigns focused on coronary heart disease awareness, there has been a decline in the proportion of SCD due to VT/VF.^1–5^ Unfortunately, there has been a simultaneous increase in pulseless electric activity (PEA) cases despite an improvement in emergency medical service (EMS) response times across the community.^6^^,^^7^

In this issue of the *European Heart Journal*, Blom and colleagues^8^ provide greater understanding of population-based factors that are associated with cardiac rhythms and outcomes after an out-of-hospital cardiac arrest (OHCA). In their surveillance study comprised of 1.85 million Dutch residents, 23 359 OHCAs were identified between 2006 and 2012. Of these, 5717 individuals received an EMS-guided resuscitation attempt. While the cohort had an equal distribution of males and females, women suffered a 15% lower overall incidence of OHCA, consistent with previous studies.^9^ In addition, the women in this analysis had a lower overall odds of survival than men. The most striking finding from this analysis is that unlike the majority of men, only 34% of women presented with a shockable cardiac rhythm. Additional analyses demonstrated similar survival rates in both sexes among the subgroup presenting with shockable rhythms.

Why are women and men manifesting OHCAs in such divergent fashions? PEA and asystole can be the late manifestation of untreated VT or VF. In the study of Blom *et al.*,^8^ there was a 4% lower rate of bystander resuscitation for women, which may have increased the likelihood of a non-shockable rhythm once the EMS had arrived. The trend of bystander inaction is consistent with prior studies.^10^ This troublesome finding may reflect inherent personal biases and reluctance on the part of a bystander (particularly men) to engage unfamiliar women, and will require continued public health campaigns to mitigate. In contrast, the responses of trained professionals, including time to EMS response or application of external defibrillators, were not influenced by sex, further suggesting that public education may be a key in closing the mortality gap.

Women consistently present with lower rates of shockable rhythms during an OHCA than men. After limiting the current analysis from The Netherlands to OHCA that occurred in public locations and controlling for resuscitation-related differences, women remain more likely than men to present with a non-shockable rhythm such as PEA or asystole during OHCA. Similar findings have been observed in the Oregon Sudden Unexpected Death Study, which identified female gender, African American race, and selected comorbidities such as pulmonary disease and syncope as risk markers for PEA compared with VF/VT.^6^ In the acute care setting, practitioners use a mnemonic that focuses on the causes of PEA by recalling the ‘Hs and Ts’ (hypovolaemia, hypoxia, tamponade, and tension pneumothorax, to name a few), but perhaps we should also consider the Xs and Ys as well. Genetics and epigenetics may help to inform part of the gap observed between men and women post-arrest. Deeper phenotyping and molecular characterization of the terminal rhythms of life may identify other clinical factors, imaging markers, and biological variability in the genetic code or human proteome that predispose a patient to either VT, VF, PEA, or asystole. Novel study designs that focus on OHCA survivors in which the presenting arrhythmia is known may allow for a thorough investigation into the factors and biological pathways implicated in shockable vs. non-shockable rhythms (*Take home figure*). This study design is clearly challenging as only a small minority of OHCA patients survive, and underlying clinical diseases and resuscitation interventions may confound the post-arrest evaluation. However, carefully designed studies that include a deeper focus on presenting rhythms at the time of an OHCA may help to maximize the potential of proteomic and genomic technologies to provide a molecular understanding similar to how they are being employed in other conditions such as atrial fibrillation and coronary artery disease.^11^

**Take home figure:**
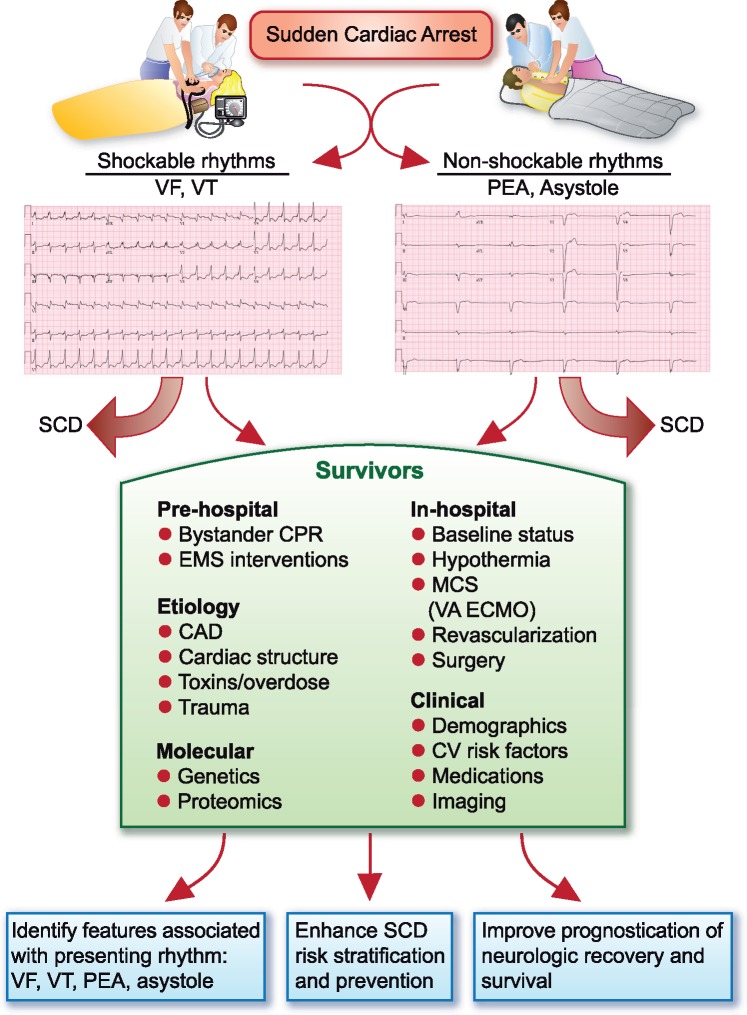
Evaluation of post-cardiac arrest patients. Novel study designs that systematically evaluate out-of-hospital cardiac arrest survivors can inform research topics related to differences in presenting rhythms at the time of sudden cardiac arrest; SCD risk stratification; and prognostication of neurologic recovery. MCS, mechanical circulatory support; VA ECMO, veno-arterial extracorporeal membrane oxygenation.

With greater disease understanding will come a greater opportunity for novel therapeutic interventions. At present, the absence of VT or VF at the time of cardiac arrest has defined how not to treat the patient. Cardiac resuscitation algorithms create broad distinctions of shockable and non-shockable; and, in large part, the non-shockable rhythms are treated as a monolithic entity. This coarse approach to clinical management is less a reflection of our belief that PEA and asystole should be treated equivalently and more emblematic of the fact that we are unsure how to parse them. While we have developed external and internal defibrillators as a successful intervention for VT and VF, meaningful interventions for other causes of SCD have remained elusive. Atropine was trialed for the management of non-shockable rhythms, but was found to be more harmful than helpful.^12^ Epinephrine increased return of spontaneous circulation, but did not improve overall survival.^13^ Some animal models have suggested a potential benefit to intentional ischaemic pre-conditioning during resuscitation, but as yet this remains unproven. Until we are able to make greater progress in the biology and risk markers for conditions such as PEA and asystole, outcomes for at-risk populations will probably remain poor.

It is conceivable that a combination of phenotypic and molecular markers may allow for a more forward approach to SCD risk stratification and targeted interventions in selected populations. Prophylactic pacemakers may be used to ward off bradycardic arrests similar to how the ICD protects from fatal arrhythmias in patients with a depressed left ventricular ejection fraction. Ultimately, SCD due to an unshockable cardiac rhythm is an evolving epidemic and a large unmet need in public health. Women in both Europe and the USA are disproportionately affected by these rhythms and have poorer clinical outcomes as a result. While genetic and epigenetic predispositions may preclude us from erasing the discrepant modes of SCD between men and women, enhanced methods of risk stratification and tailored therapies may allow us to ensure better outcomes for all.

**Conflict of interest:** none declared.

## Footnotes

† doi:10.1093/eurheartj/ehz297.

## Notes

The opinions expressed in this article are not necessarily those of the Editors of the *European Heart Journal* or of the European Society of Cardiology.

## References

[1] Cobb LA, Fahrenbruch CE, Olsufka M, Copass MK. Changing incidence of out-of-hospital ventricular fibrillation, 1980–2000. JAMA 2002;288:3008–3013. 12479765 10.1001/jama.288.23.3008

[2] Parish DC, Dinesh Chandra KM, Dane FC. Success changes the problem: why ventricular fibrillation is declining, why pulseless electrical activity is emerging, and what to do about it. Resuscitation 2003;58:31–35. 12867307 10.1016/s0300-9572(03)00104-7

[3] Polentini MS, Pirrallo RG, McGill W. The changing incidence of ventricular fibrillation in Milwaukee, Wisconsin (1992–2002). Prehosp Emerg Care 2006;10:52–60. 16418092 10.1080/10903120500366961

[4] Herlitz J, Andersson E, Bang A, Engdahl J, Holmberg M, lindqvist J, Karlson BW, Waagstein L. Experiences from treatment of out-of-hospital cardiac arrest during 17 years in Goteborg. Eur Heart J 2000;21:1251–1258. 10924315 10.1053/euhj.2000.2150

[5] Kuisma M, Repo J, Alaspaa A. The incidence of out-of-hospital ventricular fibrillation in Helsinki, Finland, from 1994 to 1999. Lancet 2001;358:473–474. 11513916 10.1016/S0140-6736(01)05634-3

[6] Teodorescu C, Reinier K, Dervan C, Uy-Evanado A, Samara M, Mariani R, Gunson K, Jui J, Chugh SS. Factors associated with pulseless electric activity versus ventricular fibrillation: the Oregon sudden unexpected death study. Circulation 2010;122:2116–2122. 21060069 10.1161/CIRCULATIONAHA.110.966333

[7] Stiell IG, Wells GA, Field BJ, Spaite DW, De Maio VJ, Ward R, Munkley DP, Lyver MB, Luinstra LG, Campeau T, Maloney J, Dagnone E. Improved out-of-hospital cardiac arrest survival through the inexpensive optimization of an existing defibrillation program: OPALS study phase II. Ontario Prehospital Advanced Life Support. JAMA 1999;281:1175–1181. 10199426 10.1001/jama.281.13.1175

[8] Blom MT, Oving I, Berdowski J, van Valkengoed IGM, Bardai A, Tan HL. Women have lower chances than men to be resuscitated and survive out-of-hospital cardiac arrest. Eur Heart J 2019;40:3824--3834. 10.1093/eurheartj/ehz297 PMC6911168 31112998

[9] Morrison LJ, Schmicker RH, Weisfeldt ML, Bigham BL, Berg RA, Topjian AA, Abramson BL, Atkins DL, Egan D, Sopko G, Rac VE, Resuscitation Outcomes Consortium Investigators. Effect of gender on outcome of out of hospital cardiac arrest in the Resuscitation Outcomes Consortium. Resuscitation 2016;100:76–81. 26705971 10.1016/j.resuscitation.2015.12.002 PMC4761304

[10] Blewer AL, McGovern SK, Schmicker RH, May S, Morrison LJ, Aufderheide TP, Daya M, Idris AH, Callaway CW, Kudenchuk PJ, Vilke GM, Abella BS, Resuscitation Outcomes Consortium (ROC) Investigators. Gender disparities among adult recipients of bystander cardiopulmonary resuscitation in the public. Circ Cardiovasc Qual Outcomes 2018;11:e004710. 30354377 10.1161/CIRCOUTCOMES.118.004710 PMC6209113

[11] Lubitz SA, Yin X, Lin HJ, Kolek M, Smith JG, Trompet S, Rienstra M, Rost NS, Teixeira PL, Almgren P, Anderson CD, Chen LY, Engstrom G, Ford I, Furie KL, Guo X, Larson MG, Lunetta KL, Macfarlane PW, Psaty BM, Soliman EZ, Sotoodehnia N, Stott DJ, Taylor KD, Weng LC, Yao J, Geelhoed B, Verweij N, Siland JE, Kathiresan S, Roselli C, Roden DM, van der Harst P, Darbar D, Jukema JW, Melander O, Rosand J, Rotter JI, Heckbert SR, Ellinor PT, Alonso A, Benjamin EJ, AFGen Consortium. Genetic risk prediction of atrial fibrillation. Circulation 2017;135:1311–1120. 27793994 10.1161/CIRCULATIONAHA.116.024143 PMC5380586

[12] Deakin CD, Morrison LJ, Morley PT, Callaway CW, Kerber RE, Kronick SL, Lavonas EJ, Link MS, Neumar RW, Otto CW, Parr M, Shuster M, Sunde K, Peberdy MA, Tang W, Hoek TL, Bottiger BW, Drajer S, Lim SH, Nolan JP, Advanced Life Support Chapter Collaborators. Part 8: Advanced life support: 2010 International Consensus on Cardiopulmonary Resuscitation and Emergency Cardiovascular Care Science with Treatment Recommendations. Resuscitation 2010;81 (Suppl 1):e93–e174. 20956032 10.1016/j.resuscitation.2010.08.027

[13] Hagihara A, Hasegawa M, Abe T, Nagata T, Wakata Y, Miyazaki S. Prehospital epinephrine use and survival among patients with out-of-hospital cardiac arrest. JAMA 2012;307:1161–1168. 22436956 10.1001/jama.2012.294

